# Supplementary material for: Spiral packing and chiral selectivity in model membranes probed by phase-resolved sum-frequency generation microscopy
Source: Nat Commun. 2024 Apr 11;15:3161. doi: 10.1038/s41467-024-47573-1 (PMC11009297; doi:10.1038/s41467-024-47573-1)
Supplement: Supplementary file 1 — Supplementary Information [file 41467_2024_47573_MOESM1_ESM.pdf]

Supplementary Information

Spiral packing and chiral selectivity in model membranes probed by phase-resolved sum-frequency generation microscopy

Alexander P. Fellows[1], Ben John[1], Martin Wolf[1], and Martin Thämer[1]*

[1]Fritz-Haber-Institute of the Max-Planck-Society, Faradayweg 4-6, 14195, Berlin, Germany

*Corresponding Author

thaemer@fhi-berlin.mpg.de

Suppl. Note 1. Singular Value Decomposition

As mentioned in the methods section, prior to Fourier transformation to convert the 3D vSFG images as a function of azimuthal sample angle to 3D images as a function of azimuthal frequencies, a Principal Component Analysis (PCA) is performed via Singular Value Decomposition (SVD) on the entire 4D dataset. This effectively groups together those spectral components that are linked throughout the entire dataset and leads to a substantial reduction of noise. The results from this SVD analysis are shown in Suppl. Fig. 1, with the single values of the first 15 SVD components shown in Suppl. Fig. 1a in decreasing order. From this, it is evident that the majority of the desired information is contained within the first two SVD components, named C1 and C2, as they are the only ones which clearly stand above the slowly decreasing baseline. This is confirmed by noting that no vibrational contrast is observed for the domains beyond the C2 component, as demonstrated in Suppl. Fig. 2.

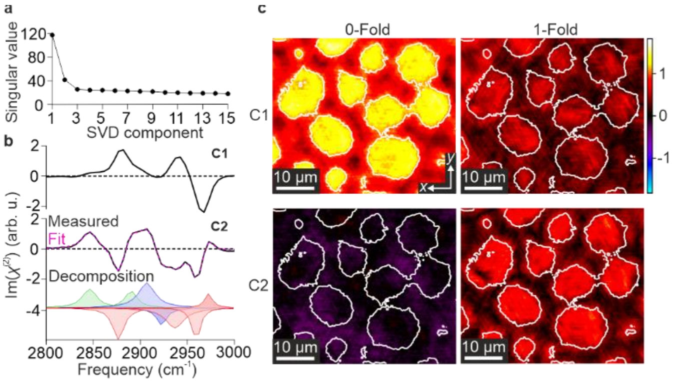

Suppl. Fig. 1 Singular Value Decomposition (SVD) and rotational analysis. a, SVD singular values (dimensionless) for the first 15 component spectra. **b**, Imaginary parts of the first (C1) and second (C2) SVD component spectra. Included is a deconvolution of the second component into its constituent bands, showing CH_2 resonances in green, CH_3 resonances in red, and unassigned bands in blue. **c**, Images of the first two SVD components at the 0-fold and 1-fold azimuthal frequencies. As the 0-fold images are entirely real by definition, the real part of the complex FT is shown for each component, whereas the magnitudes are shown for the 1-fold images as these are generally complex. All images are shown on the same colour scale given in arbitrary units. White contours from the 0-fold C1 image are shown in all images to highlight the domain locations.

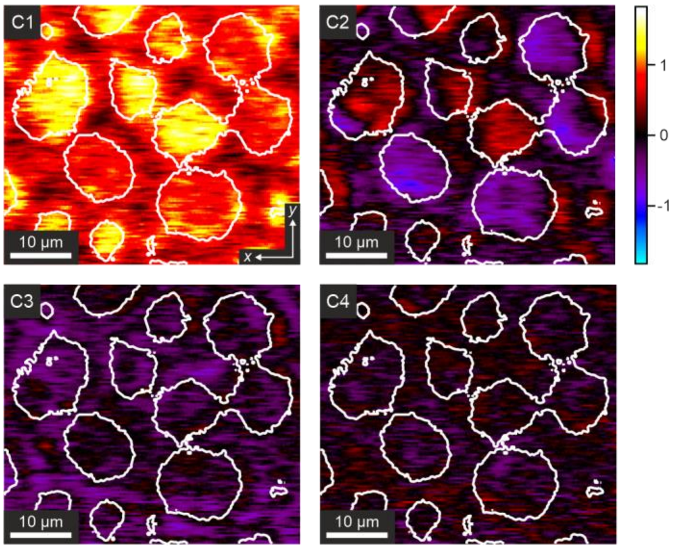

Suppl. Fig. 2 Singular Value Decomposition (SVD) component images. SVD images of the first four SVD components, C1-C4, for the 0° sample rotation angle. All images are shown on the same colour scale given in arbitrary units. White contours from the 0-fold C1 image (Suppl. Fig. 1c) are shown in all images to highlight the domain locations.

The spectra of the C1 and C2 components are given in Suppl. Fig. 1b, only presenting the imaginary (absorptive) parts, where the C2 component is also deconvoluted into its constituent resonances. By comparison to the in-plane and out-of-plane spectra shown in Fig. 3b (main text), it is clear that remarkable similarity exists between these and the SVD component spectra, as expected. Specifically, the C1 component largely indistinguishable from the out-of-plane contribution and the C2 component shows a very similar line-shape and deconvolution profile to the in-plane contribution. This similarity is further confirmed in the rotational analysis of the SVD components given in Suppl. Fig. 1c which clearly shows the C1 component to be dominated by its 0-fold contribution and similarly the C2 component dominated by its 1-fold contribution.

It is noteworthy, however, that the C1 component does show some (albeit small compared to the 0-fold) intensity at the 1-fold azimuthal frequency, indicating that the SVD has not completely isolated the in-plane and out-of-plane spectra. Instead, they can be generated from a combination of the C1 and C2 components. This is demonstrated in Suppl. Fig. 3 which overlaps both the in-plane and out-of-plane spectra from Fig. 3b (main text) with combinations of the C1 and C2 spectra, showing excellent agreement for both. The out-of-plane spectra is thus generated from a combination of C1 and C2 in roughly a 10:1 ratio, demonstrating clear dominance from the former contribution, and the in-plane spectra from a 5:9 ratio, showing more similar values but still majoritively being described by the C2 component. Nevertheless, as the C2 component is predominantly an in-plane contribution, its rotational phase can be used as a direct measure of the in-plane molecular directionality.

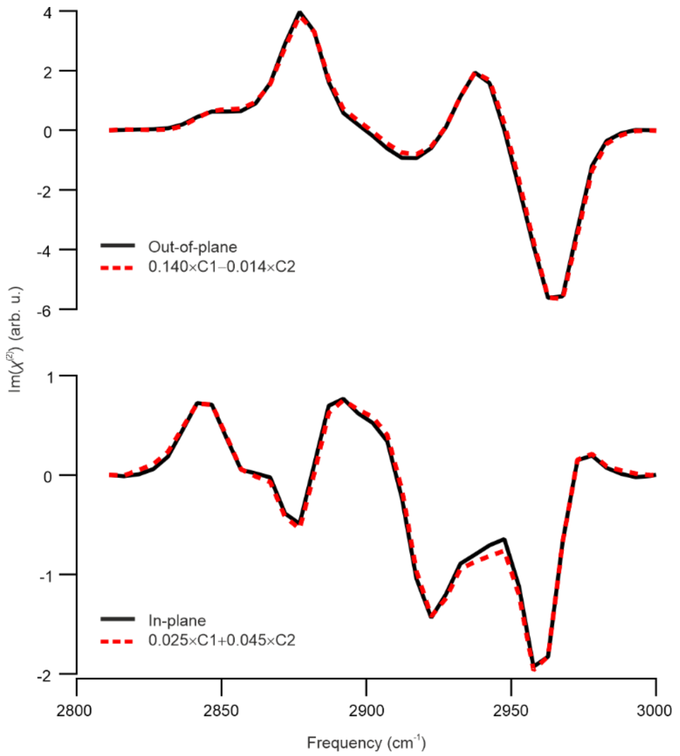

Suppl. Fig. 3 Comparing SVD component spectra with in-plane and out-of-plane contributions. Spectra obtained from the 0-fold (out-of-plane) and 1-fold (in-plane) azimuthal frequencies (solid black) compared to combinations of the first two SVD component spectra (dashed red).

Suppl. Note 2. Determining the In-Plane Molecular Direction

As noted above, the C2 SVD component can be used to access the in-plane molecular direction. This is achieved by extracting the rotational phase at the 1-fold azimuthal frequency from the complex Fourier transform of the 3D dataset (pixel vs. azimuthal sample angle) for the C2 component. The direction of this phase is then defined based on the coordinate system in the Laboratory frame, defined with the incident beams be directed within the *xz*-plane towards the positive *x*-direction, which coincides with the coordinate system of the sample frame at 0°.

In order to convert this direction of the C2 component into a specific direction based on the molecular structure, one can then utilise the C2 component spectrum, as shown in Suppl. Fig. 1b. Given that the symmetric CH_3 resonance has a very well-defined transition dipole direction and is fairly spectrally isolated, this provides a good marker on which to base the molecular direction. In the C2 spectrum, it can then be observed that the symmetric CH_3 stretch has a negative sign. This indicates that the direction of the overall C2 component must be directed opposite to the in-plane contribution of the CH_3 symmetric stretch transition dipole. This is the in-plane molecular direction defined in Figs. 1 and 4 (main text).

Suppl. Note 3. Rotationally-Dependent vSFG

As discussed in the main text and indicated in Fig. 3, the in-plane molecular orientations are determined from a rotational analysis procedure whereby vSFG images are recorded as a function of azimuthal angle, φ, and subjected to a complex Fourier transform. This converts the azimuthal axis of the 4D dataset to azimuthal (rotational) frequencies, f, as shown schematically in Suppl. Fig. 4.

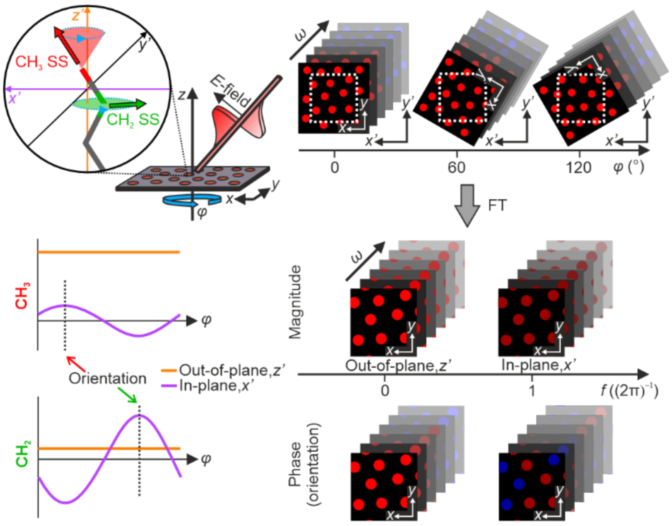

Suppl. Fig. 4 Rotational analysis procedure. Schematic of the rotational analysis procedure showing the variation in CH_2 and CH_3 transition dipoles upon rotation and the modulation of their projection along the probed x' and z' directions. Also shown are representations of the four-dimensional dataset that is generated, with SFG microscopy images at different spectral frequencies (ω) recorded at multiple sample rotations (φ), and their conversion into azimuthal frequencies, f, via a Fourier transform (FT), yielding both magnitude and phase data at each azimuthal and spectral frequency.

By considering the projection of a specific transition dipole vector onto the probed x' and z' axes, rotation of the sample clearly modulates the in-plane component sinusoidally but leaves the out-of-plane component unchanged. This is also represented in Suppl. Fig. 4 for the CH_2

SS and CH_3 SS. This shows that the former (being mostly in-plane) has only a small, constant out-of-plane contribution and a much larger in-plane contribution which oscillates with rotation. By contrast, the CH_3 group is mostly directed out-of-plane and thus has a large, constant out-of-plane contribution along with a much smaller, although still non-negligible, contribution from its in-plane projection. Also indicated are phase shifts of the sinusoidal oscillations of the in-plane projections for both modes, demonstrating that the molecular orientation is encoded in the phase of the 1-fold azimuthal frequency.

Suppl. Note 4. Theoretical Considerations for Rotational vSFG

The theoretical details of SFG and general nonlinear optics have been widely reviewed and can be found elsewhere in the literature.[1–4] In the following section, the specific details underlying the rotational dependency of the observed signals are briefly summarised to demonstrate how they can be used to decipher the molecular directionality in heterogeneous systems like those studied in this work.

SFG, as a second-order optical process, is generated through the non-linear coupling of two fields through the second-order susceptibility of the species being probed, $\chi^{(2)}$. When probing in the PPP polarisation combination, as done here, the incident and output fields are all directed in the *xz*-plane and thus probe the eight components of $\chi^{(2)}$ listed in Eq. 1.

$$\chi^{(2)}_{XXX}, \chi^{(2)}_{XXZ}, \chi^{(2)}_{XZX}, \chi^{(2)}_{ZXX}, \chi^{(2)}_{XZZ}, \chi^{(2)}_{ZXZ}, \chi^{(2)}_{ZZX}, \chi^{(2)}_{ZZZ} \tag{1}$$

For systems that exhibit in-plane rotational isotropy, components with linear or cubic *x*-dependency must vanish, thus removing half of the contributions. For heterogeneous systems like those studied in this work, however, no such symmetry exists and thus all components can potentially be non-zero. Nevertheless, when employing a collinear beam geometry (as done here), half of these contributions cancel due to the symmetry in the off-resonant polarisability, as in Eq. 2, and their opposing signs in the effective PPP susceptibility.

$$\chi^{(2)}_{xzx} = \chi^{(2)}_{zxx}; \chi^{(2)}_{xzz} = \chi^{(2)}_{zxz} \tag{2}$$

This leaves four contributions, namely the *xxx*, *xxz*, *zzx*, and *zzz*. These macroscopic, sample-frame susceptibilities can then be described by the molecular hyperpolarisabilities by use of a coordinate transformation using three Euler angles which completely describe the specific orientation of any functional group, namely the tilt angle, θ, the in-plane rotation angle, ϕ, and the twist angle, ψ, as given by Eq. 3.

$$\begin{pmatrix} x \\ y \\ z \end{pmatrix} = \begin{pmatrix} -\cos\psi\sin\phi - \cos\theta\cos\phi\sin\psi & \sin\psi\sin\phi - \cos\theta\cos\phi\cos\psi & \sin\theta\cos\phi \\ \cos\psi\cos\phi - \cos\theta\sin\phi\sin\psi & -\sin\psi\cos\phi - \cos\theta\sin\phi\cos\psi & \sin\theta\sin\phi \\ \sin\theta\sin\psi & \sin\theta\cos\psi & \cos\theta \end{pmatrix} \begin{pmatrix} a \\ b \\ c \end{pmatrix} \tag{3}$$

By considering the methyl symmetric stretch (which is used in this work to define the in-plane molecular direction), the local C_S symmetry of the CH_3 group (as only a minor perturbation to C_{3V} symmetry) results in only three contributing hyperpolarisability components, namely β_{aac}, β_{bbc}, and β_{ccc}, where $\beta_{aac} = \beta_{bbc} = R\beta_{ccc}$, with R relating to the Raman polarisability ratio. With this relation, the four susceptibility components can be expressed as in Eqs. 4-7, where the azimuthal rotation angle, φ has also been included as modulation to the Euler angle, ϕ.

$$\chi_{xxx}^{(2)}(CH_3SS) = \beta_{ccc}[R\sin\theta\cos(\phi+\varphi)\,(\sin^2(\phi+\varphi)+\cos^2\theta\cos^2(\phi+\varphi)) + \sin^3\theta\cos^3(\phi+\varphi)] \quad (4)$$

$$\chi_{xxz}^{(2)} = \beta_{ccc}[R\cos\theta(\sin^2(\phi+\varphi)+\cos^2\theta\cos^2(\phi+\varphi)) + \sin^2\theta\cos\theta\cos^2(\phi+\varphi)] \quad (5)$$

$$\chi_{zzx}^{(2)} = \beta_{ccc}[R\sin^3\theta + \sin\theta\cos^2\theta]\cos(\phi+\varphi) \quad (6)$$

$$\chi_{zzz}^{(2)} = \beta_{ccc}[R\sin^2\theta\cos\theta + \cos^3\theta] \quad (7)$$

Taking a Fourier transform of the azimuthal rotational dependency coverts these contributions into their dependencies on the azimuthal frequency, f, leads to Eqs. 8-11.

$$\mathcal{F}\left[\chi_{xxx}^{(2)}\right](f) = \beta_{ccc}\Big[R\sin\theta\left(-\frac{1}{4}e^{-3i\phi}\delta(f-3)+\frac{1}{4}e^{-i\phi}\delta(f-1) + \cos^2\theta\left(\frac{1}{4}e^{-3i\phi}\delta(f-3)+\frac{3}{4}e^{-i\phi}\delta(f-1)\right)\right) + \sin^3\theta\left(\frac{1}{4}e^{-3i\phi}\delta(f-3)+\frac{3}{4}e^{-i\phi}\delta(f-1)\right)\Big] \quad (8)$$

$$\mathcal{F}\left[\chi_{xxz}^{(2)}\right](f) = \beta_{ccc}\Big[R\cos\theta\left(-\frac{1}{2}e^{-2i\phi}\delta(f-2)+\frac{1}{2}\delta(f) + \cos^2\theta\left(\frac{1}{2}e^{-2i\phi}\delta(f-2)+\frac{1}{2}\delta(f)\right)\right) + \sin^2\theta\cos\theta\left(\frac{1}{2}e^{-2i\phi}\delta(f-2)+\frac{1}{2}\delta(f)\right)\Big] \quad (9)$$

$$\mathcal{F}\left[\chi_{zzx}^{(2)}\right](f) = \beta_{ccc}[R\sin^3\theta + \sin\theta\cos^2\theta]e^{-i\phi}\delta(f-1) \quad (10)$$

$$\mathcal{F}\left[\chi_{zzz}^{(2)}\right](f) = \beta_{ccc}[R\sin^2\theta\cos\theta + \cos^3\theta]\delta(f) \quad (11)$$

With this description, the Fourier transform of the total PPP response can be written in terms of its azimuthal frequency contribution. The components at the 0-fold and 1-fold azimuthal frequencies, f_0 and f_1, respectively, are then given by Eqs. 11 and 12, where the weighting coefficients c_1-c_4 are governed by the incidence angle and Fresnel factor corrections.

$$\chi_{PPP}^{(2)}(f_0) = \beta_{ccc}\cos\theta\left[\frac{1}{2}c_1(R(1+\cos^2\theta)+\sin^2\theta) + c_2(R\sin^2\theta+\cos^2\theta)\right] \quad (12)$$

$$\chi_{PPP}^{(2)}(f_1) = \beta_{ccc}\sin\theta\left[\frac{1}{4}c_3(R(1+3\cos^2\theta)+3\sin^2\theta) + 4c_4(R\sin^2\theta+\cos^2\theta)\right]e^{-i\phi} \quad (13)$$

These equations clearly demonstrate that the 0-fold frequency component is only dictated by the tilt angle of the molecule and thus solely describes the out-of-plane contribution. On the other hand, the 1-fold frequency component is modulated by both the tilt angle and rotation angle. Importantly, however, the tilt angle only modulates the magnitude and the rotation angle only the phase of the response. Specifically, it is clear that the phase of the response is exactly equivalent to the opposite of the in-plane direction of the transition dipole. Hence, using the specific Euler transformation defined in Eq. 3 and by defining the in-plane orientation of the molecule as opposite of the in-plane CH_3 response, as done here, the phase of the 1-fold response directly corresponds to the in-plane direction. Clearly, despite the response being non-linear, a linear treatment based on the 1-fold frequency contribution is perfectly valid for determining the in-plane molecular directionality.

Suppl. Note 5. Appearance of the Spectra and Out-of-Plane Molecular Orientation

As discussed in the main text, for lipids in an upright orientation (tails 'pointing up'), the expected responses in the spatially averaged PPP spectrum with a 36° incidence angle are positive for the symmetric stretch (SS) and negative for the antisymmetric stretch (AS). The origin of this expectation is, however, far from trivial as the PPP response contains multiple tensor components which can interfere. In the following, the contributions from each resonance are defined based on an Euler transformation of their molecular hyperpolarisabilities (using the transformation given in Eq. 3 above) and shown using simulations to yield the expected signs in the overall PPP response. Thereafter, the observed spatially averaged PPP spectrum is decomposed into its constituent tensor components, highlighting that it is indeed consistent with the expected 'pointing up' out-of-plane structure.

As noted in the theory outlined above, the PPP response in a collinear beam geometry contains four non-cancelling tensor components, namely *xxx*, *xxz*, *zzx*, and *zzz*. When averaging the responses across the entire image, however, the *xxx* and *zzx* responses will largely cancel owing to their intrinsic symmetry properties. This leaves the *xxz* and *zzz* contributions which are typically discussed in spectroscopy measurements under the assumption of in-plane isotropy. Under this approximation, the relevant tensor components for the symmetric and antisymmetric stretching modes (taking the CH_3 group to have approximately C_{3v} symmetry) are given by Eqs. 14-17, having integrated over the Euler angles ϕ and ψ.

For the symmetric stretch (SS):

$$\chi^{(2)}_{xxz} = \chi^{(2)}_{yyz} = \frac{1}{2}\beta_{ccc}\cos\theta\,[R(1+\cos^2\theta)+\sin^2\theta] \quad (14)$$

$$\chi^{(2)}_{zzz} = \beta_{ccc}\cos\theta\,[R\sin^2\theta+\cos^2\theta] \quad (15)$$

For the antisymmetric stretch (AS):

$$\chi^{(2)}_{xxz} = \chi^{(2)}_{yyz} = -\beta_{aca}\cos\theta\sin^2\theta \quad (16)$$

$$\chi^{(2)}_{zzz} = 2\beta_{aca}\cos\theta\sin^2\theta \quad (17)$$

If the methyl tilt angle, θ, is within 90° of the surface normal (i.e., the tail is pointing 'up'), $\cos\theta > 0$ and thus the sign of the responses is purely dictated by the sign of the molecular hyperpolarisability components (which are the same[5]). On noting that the PPP responses from the CH_3 groups of self-assembled monolayers (SAMs) on gold appear as dips from the strong non-resonant background which has a well-known positive phase[4], it is clear that *zzz* contributions which dominate the PPP response on metals must be negative for the pointing 'up' SAM geometry. Therefore, here, the SS will appear as dips in both *xxz* and *zzz* whereas the AS will be a dip in *zzz* but a peak in *xxz*. As the PPP response is essentially given by *zzz* – *xxz* (as shown in Eq. 18, including Fresnel factors and the incidence angle, θ_i), it is thus clear that the AS contributions will constructively interfere and form a pronounced dip whereas the two SS contributions will destructively interfere, leaving the sign of the SS ill-defined and dependent on the experimental settings and molecular tilt angle.

$$\chi^{(2)}_{PPP} = \sin^3\theta_i\, L_z(\omega_3)L_z(\omega_2)L_z(\omega_1)\chi^{(2)}_{zzz} - \sin\theta_i\cos^2\theta_i\, L_x(\omega_3)L_x(\omega_2)L_z(\omega_1)\chi^{(2)}_{xxz} \quad (18)$$

For the SS, the overall susceptibility is thus given in terms of its hyperpolarisabilities by Eq. 19, having grouped together the tilt-angle and hyperpolarisability ratio dependency into a prefactor, *P*.

$$\chi^{(2)}_{PPP}(SS) = L_z(\omega_1)\sin\theta_i\,\beta_{ccc}\cos\theta\,[\sin^2\theta_i L_z(\omega_3)L_z(\omega_2)(R\sin^2\theta + \cos^2\theta) - \frac{1}{2}\cos^2\theta_i\,L_x(\omega_3)L_x(\omega_2)(R(1+\cos^2\theta)+\sin^2\theta)] = PL_z(\omega_1)\beta_{ccc} \quad (19)$$

The sign of the SS resonance can thus be determined by simulating the prefactor, P, for different tilt angles from 0-90° and for different possible values for R (which is defined between 1 and 4[5]). This simulation is shown in Suppl. Fig. 5, where it is clear that the prefactor is always negative for a 'pointing up' methyl group. This response will hence have the opposite sign to the overall AS (which is negative) and thus present as a peak in the spectrum. The overall PPP response is then expected to appear with peaks for the SS and its FR and a dip for the AS.

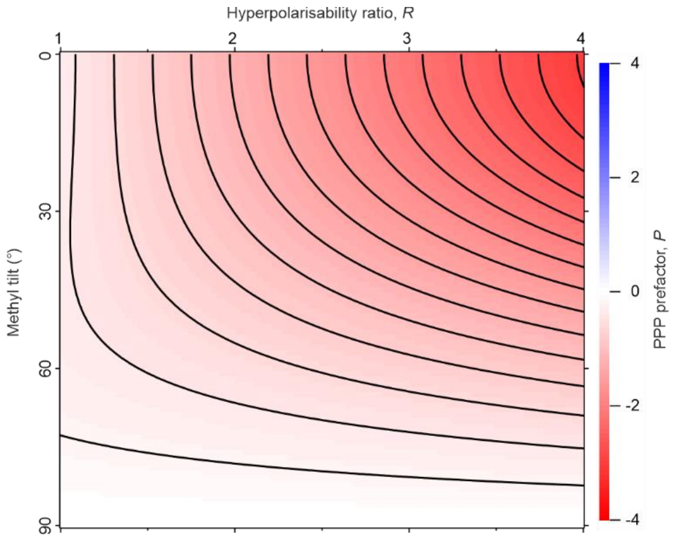

Suppl. Fig. 5 Simulated PPP prefactor. Simulation of the prefactor, P (dimensionless), as defined in Eq. 19, for the CH_3 SS in the overall PPP response as a function of the methyl tilt angle and hyperpolarisability ratio, R (dimensionless).

As the observed PPP spectrum contains the *xxz* and *zzz* contributions, they can be individually extracted by measuring the SSP response which only contains a single tensor element when spatially averaging the responses, namely the *yyz*(=*xxz*) component, as in Eq. 20.

$$\chi^{(2)}_{SSP} = \sin\theta_i\,L_y(\omega_3)L_y(\omega_2)L_z(\omega_1)\chi^{(2)}_{yyz} \quad (20)$$

The overall SSP spectrum is presented in Suppl. Fig. 6 (green) and shows a 'dip-dip-peak' line-shape that is unambiguously characteristic of a 'pointing up' tail orientation, as noted above. With this response, the two surviving components to the overall PPP spectrum can be extracted based on Eqs. 21 and 22, with the decomposed spectrum shown in Suppl. Fig. 6 (overall: black, -*xxz*: blue, *zzz*: red).

$$\sin^3\theta_i\,L_z(\omega_3)L_z(\omega_2)L_z(\omega_1)\chi^{(2)}_{zzz} = \chi^{(2)}_{PPP} + \cos^2\theta_i\frac{L_x(\omega_3)L_x(\omega_2)}{L_y(\omega_3)L_y(\omega_2)}\chi^{(2)}_{SSP} \quad (21)$$

$$-\sin\theta_i\cos^2\theta_i\,L_x(\omega_3)L_x(\omega_2)L_z(\omega_1)\chi^{(2)}_{xxz} = -\cos^2\theta_i\frac{L_x(\omega_3)L_x(\omega_2)}{L_y(\omega_3)L_y(\omega_2)}\chi^{(2)}_{SSP} \quad (22)$$

With the *xxz* and *zzz* contributions isolated, it is clear that they do indeed show the expected line-shapes for a 'pointing up' orientation and thus the overall PPP spectrum is consistent with this out-of-plane molecular structure.

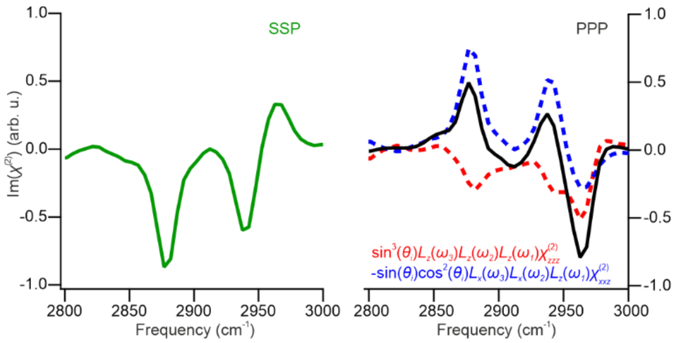

Suppl. Fig. 6 Spatially averaged SSP and PPP vSFG spectra. vSFG spectra averaged over the entire microscope image in the SSP (green) and PPP (black) polarisation combinations. The PPP spectrum has been broken down into its two main constituents: *zzz* (red) and *-xxz* (blue).

Suppl. Note 6. Further Arrow Representations

The in-plane directionality presented in Fig. 4 in the main text shows the full phase map for each enantiomer along with arrow representations for a selected domain to analyse their molecular packing structures. Suppl. Fig. 7 presents the same phase maps as in Fig. 4b (main text) alongside their corresponding arrow representations for each domain within the images. As stated in the main text, it is clear that, whilst there are differences between the domains within each sample, they all show analogous packing structures, disregarding some domains which clearly possess structural defects that likely arise from coalescence or growth abnormalities. Specifically, for the (*R*)-enantiomer, each domain possesses a general clockwise turning direction, but only shows modest curvature. By contrast, the domains in the (*S*)-enantiomer sample clearly show full spiral structures with anticlockwise turning directions. Specifically, this highlights that the spiralling structure and affinity to form with the spiral centre inside or outside of the domains is common to each well-formed domain and thus represents an intrinsic property of sample associated with their molecular composition.

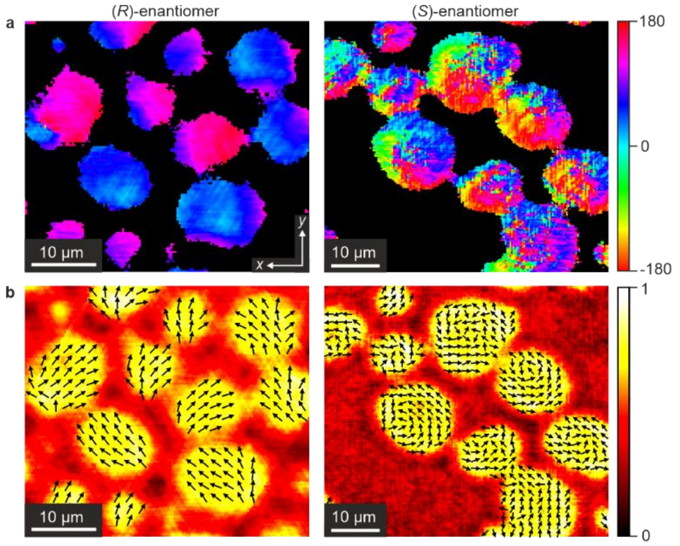

Suppl. Fig. 7 Phase and arrow representations of domains. a, Images showing the in-plane phase direction maps as in Fig. 4 (main text) according the presented colour scale representing angles of -180 to 180° relative to the *x*-direction, as for the colour wheel given in Fig. 4b (main text). **b**, Corresponding molecular direction arrow representations for each domain in the image. Each arrow represents the average phase for a 6×6 pixel region and are shown on top of the normalised 0-fold magnitude image, given by the presented colour scale.

Suppl. Note 7. Domain Density and Orientational Order Calculations

As discussed in the main text, the (*R*)-(*R*) and (*S*)-(*R*) lipid mixtures clearly show significant differences in their in-plane molecular packing. This implies vastly different circumferential line tensions that are likely connected to changes in their formation and growth processes. A further way of assessing these structural differences is from their vSFG responses and relative surface coverages. On inspection of the wider vSFG images presented in Fig. 4a (main text), it becomes evident that the surface coverage of the LC phase in the (*S*)-(*R*) mixture is significantly lower than for the (*R*)-(*R*) mixture, despite both containing the same ratio of DPPC to POPC and being formed under equal conditions. This clearly indicates that the formation process of the LC domains must possess some enantioselectivity. As for the comparison of their vSFG responses, these are related to their second-order susceptibilities which represent the macroscopic average of the molecular hyperpolarisabilities. The overall signals are thus dictated by the specific average orientation in the lab-frame, the molecular surface density, *N*, and the degree of orientational cancellation of individual molecular responses, defined here by introducing parameter, *O*, between 0 and 1, indicating full and no cancellation, respectively. As the observed signals effectively arise only from the DPPC tails, the molecular hyperpolarisability can be taken to be constant within the molecular frame, thus only presenting its orientation-dependent projections in the lab-frame. Any variation in the observed signals can thus be described by a change in any combination of these three parameters.

Suppl. Table 1 Parameters used for the Fresnel factor and beam geometry corrections.

Parameter	Value
n_{air}	1
$n_{\text{FS, 3450 nm}}$	1.4074
$n_{\text{FS, 690 nm}}$	1.4555
$n_{\text{FS, 575 nm}}$	1.4589
n_{lipid}	1.18
Monolayer thickness, *h* (nm)	2
$L_{xx,\text{ 3450 nm}}$	1.0104
$L_{xx,\text{ 690 nm}}$	1.0223
$L_{xx,\text{ 575 nm}}$	1.0232
$L_{zz,\text{ 3450 nm}}$	0.7989
$L_{zz,\text{ 690 nm}}$	0.8083
$L_{zz,\text{ 575 nm}}$	0.8090
Incident angle, θ_i (°)	36

In order to make a meaningful comparison, any orientation-dependence must be removed by extracting the effective magnitude of the response. This can be obtained from the sum-of-squares of the *x*, *y*, and *z* contributions that are accessible using the 0-fold and 1-fold azimuthal frequencies. Here, this analysis is performed using the CH_3 symmetric stretch which, due to the mostly upright lipid structure, is dominated by its *z*-response. It is thus assumed that the 0-fold and 1-fold contributions are dominated by the *zzz* and *zzx* (/*zzy*) components of the second-

order susceptibility, respectively. These responses can thus be corrected for the specific Fresnel factors and incident beam angle, as given in Suppl. Table 1, to remove any experimental influence and isolate the intrinsic *zzz* and *zzx* (/*zzy*) terms. The effective magnitudes then become the square-root of the sum-of-squares of these contributions. Any change in these magnitudes thus can only be described by a change in molecular density, *N*, or the orientational cancellation parameter, *O*.

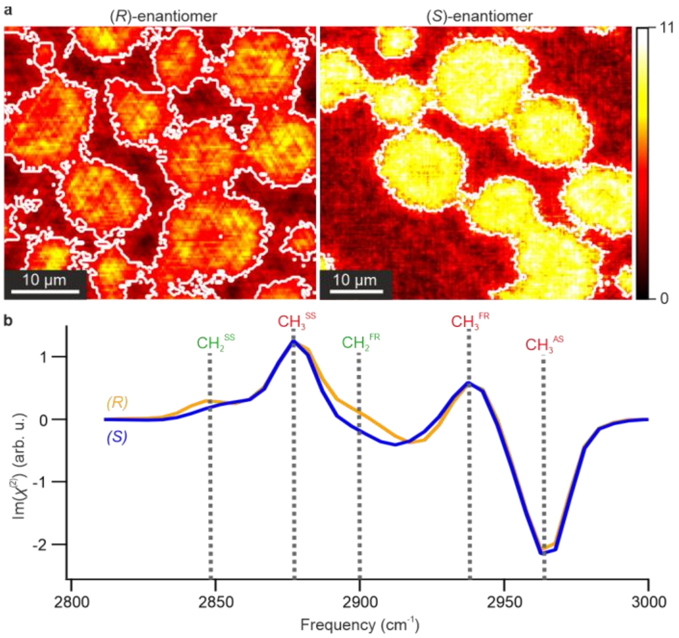

Suppl. Fig. 8 Order and density of the condensed domains. a, Images of the symmetric methyl stretch for both enantiomers, corrected for Fresnel factors and beam geometry, represented as the effective magnitude of the transition dipole and both shown on the same colour scale given in arbitrary units. **b**, Imaginary part of the C1 component spectra for both enantiomers ((*R*) – orange, (*S*) – blue) with the main resonances highlighted.

Suppl. Table 2 Determined areas and vSFG magnitude parameters from the images in Suppl. Fig. 8a.

Parameter	(*R*)-enantiomer	(*S*)-enantiomer
A_{T} (pixel)	19932	33485
A_{LC} (pixel)	11561	14399
A_{LE} (pixel)	8371	19086
$A_{\mathrm{LC}}N_{\mathrm{LC}}O_{\mathrm{LC}}$ (arb. u.)	70432	128996
$A_{\mathrm{LE}}N_{\mathrm{LE}}O_{\mathrm{LE}}$ (arb. u.)	29384	75272
$N_{\mathrm{LC}}O_{\mathrm{LC}}$ (arb. u.)	6.09	8.96
$N_{\mathrm{LE}}O_{\mathrm{LE}}$ (arb. u.)	3.51	3.94

Suppl. Fig. 8a shows the resulting magnitude maps for both enantiomer samples, where the (*S*)-enantiomer clearly yields a stronger response for the LC domains. This shows that this enantiomeric mixture must form domains with an increased DPPC density and/or decreased orientational cancellation (larger *O*). To determine the origin of this observed difference, certain parameters can be extracted from the magnitude images in Suppl. Fig. 8a. Specifically, the total surface area, A_{T}, along with the corresponding area coverages of each phase, A_{LC} and A_{LE}, are determined. Thereafter, the vSFG magnitude images in Suppl. Fig. 8a are integrated over each phase, which must yield the product of the area, number density, and the orientation

parameter 'O', i.e., $A_{LC}N_{LC}O_{LC}$ and $A_{LE}N_{LE}O_{LE}$ (effectively treating the hyperpolarisability as unity and neglecting it from the expressions). These parameters are given in Suppl. Table 2, also normalising the integrated magnitudes based on the areas to yield $N_{LC}O_{LC}$ and $N_{LE}O_{LE}$.

With these parameters in hand, it becomes possible to estimate the ratios of N_{LC} and O_{LC} for the two enantiomers using some simple assumptions. Firstly, it is assumed that the average number density of DPPC across the entire surface (i.e., in both phases) is the same for both enantiomers. This can be summarised mathematically as in Eq. 23. This is a very reasonable assumption as both samples were generated with the same ratio of DPPC to POPC at identical surface pressures, as mentioned previously.

$$\frac{N_{\mathrm{LE}}^{R}A_{\mathrm{LE}}^{R}+N_{\mathrm{LC}}^{R}A_{\mathrm{LC}}^{R}}{A_{\mathrm{T}}^{R}}=\frac{N_{\mathrm{LE}}^{S}A_{\mathrm{LE}}^{S}+N_{\mathrm{LC}}^{S}A_{\mathrm{LC}}^{S}}{A_{\mathrm{T}}^{S}} \tag{23}$$

Secondly, it is also assumed that the orientation parameter in the liquid-expanded (LE) phase is the same for both enantiomers, i.e., as in Eq. 24. Again, this assumption is reasonable since the LE phase yields only small vSFG contributions and is generally considered to be largely structurally disordered and dominated by POPC.

$$O_{\mathrm{LE}}^{R}=O_{\mathrm{LE}}^{S} \tag{24}$$

With these two assumptions, the parameters in Suppl. Table 2 can be analysed, where the number density ratio in the LE phase can be explicitly determined, as in Eq. 25.

$$\frac{N_{\mathrm{LE}}^{R}}{N_{\mathrm{LE}}^{S}}=\frac{N_{\mathrm{LE}}^{R}O_{\mathrm{LE}}^{R}}{N_{\mathrm{LE}}^{S}O_{\mathrm{LE}}^{S}}=\frac{3.51}{3.94}=0.89 \tag{25}$$

Similarly, the values for the areas can be implemented in Eq. 23 to yield Eq. 26.

$$0.42N_{\mathrm{LE}}^{R}+0.58N_{\mathrm{LC}}^{R}=0.57N_{\mathrm{LE}}^{S}+0.43N_{\mathrm{LC}}^{S} \tag{26}$$

Then, utilising Eq. 25, this can be rearranged to yield Eqs. 27 and 28.

$$N_{\mathrm{LC}}^{S}=\frac{\left(0.42-\frac{0.57}{0.89}\right)N_{\mathrm{LE}}^{R}+0.58N_{\mathrm{LC}}^{R}}{0.43} \tag{27}$$

$$\frac{N_{\mathrm{LC}}^{S}}{N_{\mathrm{LC}}^{R}}=1.35-0.51\frac{N_{\mathrm{LE}}^{R}}{N_{\mathrm{LC}}^{R}} \tag{28}$$

To obtain the desired quantity on the left side of Eq. 28, the number density ratio between the LE and LC phase needs to be determined. Given that the LC phase is known to be DPPC-rich and the LE phase DPPC-poor, clearly this value is substantially less than 1. Nevertheless, as indicated in the main text, there are still spectral contributions from DPPC in the LE phase, showing that this ratio is also non-zero. As a way of achieving a good estimate of the exact value, we can turn to the vSFG responses from both phases. The spectra averaged across each phase presented in Fig. 2c (main text, LC in red and LE in blue) show a relative amplitude ratio of the CH_3 SS of $\approx$0.28. As these spectra are spatially averaged (within their respective regions), they are dominated by the out-of-plane response, and thus combine the molecular density of DPPC with the orientation parameter in the z direction. By comparing the vSFG responses from pure DPPC and POPC monolayers given elsewhere in the literature[6], along with their molecular densities, we find that the relative out-of-plane orientation parameters between the two lipids are almost equal. Therefore, assuming that any DPPC present in the LE POPC-dominated phase has a similar order to POPC, it hence becomes reasonable to take this value of 0.28 as an estimate of the relative DPPC densities.

This then yields the density ratio between the (*S*)- and (*R*)-enantiomers as in Eq. 29.

$$\frac{N_{\mathrm{LC}}^{S}}{N_{\mathrm{LC}}^{R}} = 1.21 \tag{29}$$

Clearly, therefore, the (*S*)-enantiomer sample has LC domains with a greater DPPC density. Using this value, the ratio of orientation parameters for the LC phase between the two enantiomers can also be determined, as in Eq. 30.

$$\frac{O_{\mathrm{LC}}^{S}}{O_{\mathrm{LC}}^{R}} = \frac{N_{\mathrm{LC}}^{S} O_{\mathrm{LC}}^{S}}{N_{\mathrm{LC}}^{R} O_{\mathrm{LC}}^{R}} \times \frac{N_{\mathrm{LC}}^{R}}{N_{\mathrm{LC}}^{S}} = 1.22 \tag{30}$$

Clearly this suggests that the (*S*)-enantiomer domains are both more DPPC-rich (higher density) and more ordered (less orientational cancellation), compared to the (*R*)-enantiomer domains.

Using these values for the relative densities of DPPC in each phase (Eqs. 25 and 30), we can then further analyse the data to determine the relative densities of POPC in the LC phase, hereafter defined as $\widehat{N}_{\mathrm{LC}}^{R/S}$. For this, we make two further assumptions. Firstly, we assume that the average number density for POPC is the same for both enantiomeric mixtures, as described by Eq. 31.

$$\widehat{N}_{\mathrm{T}}^{R} = \frac{\widehat{N}_{\mathrm{LE}}^{R} A_{\mathrm{LE}}^{R} + \widehat{N}_{\mathrm{LC}}^{R} A_{\mathrm{LC}}^{R}}{A_{\mathrm{T}}^{R}} = \frac{\widehat{N}_{\mathrm{LE}}^{S} A_{\mathrm{LE}}^{S} + \widehat{N}_{\mathrm{LC}}^{S} A_{\mathrm{LC}}^{S}}{A_{\mathrm{T}}^{S}} = \widehat{N}_{\mathrm{T}}^{S} \tag{31}$$

This follows logically from the same assumption for DPPC expressed in Eq. 23. Secondly, we assume that the total number density of both lipids in the LE phase is also the same for both mixtures, as in Eq. 32.

$$N_{\mathrm{LE}}^{R} + \widehat{N}_{\mathrm{LE}}^{R} = N_{\mathrm{LE}}^{S} + \widehat{N}_{\mathrm{LE}}^{S} \tag{32}$$

This is a reasonable assumption given that similar molecular packing is expected in the LE phase which is known to be dominated by POPC.

The density of POPC in the LC phase can be written in terms of the total, averaged surface density and that in the LE phase as in Eq. 33 for the (*R*)-enantiomer.

$$\widehat{N}_{\mathrm{LC}}^{R} = \frac{\widehat{N}_{\mathrm{T}}^{R} A_{\mathrm{T}}^{R} - \widehat{N}_{\mathrm{LE}}^{R} A_{\mathrm{LE}}^{R}}{A_{\mathrm{LC}}^{R}} \tag{33}$$

Then, we make use of the known ratio of DPPC to POPC (4:1), such that $\widehat{N}_{\mathrm{T}}^{R} = \frac{1}{4} N_{\mathrm{T}}^{R}$ and $\widehat{N}_{\mathrm{LC}}^{R}$ can be written as in Eq. 34.

$$\widehat{N}_{\mathrm{LC}}^{R} = \frac{N_{\mathrm{LE}}^{R} A_{\mathrm{LE}}^{R} + N_{\mathrm{LC}}^{R} A_{\mathrm{LC}}^{R}}{4 A_{\mathrm{LC}}^{R}} - \widehat{N}_{\mathrm{LE}}^{R} \frac{A_{\mathrm{LE}}^{R}}{A_{\mathrm{LC}}^{R}} \tag{34}$$

Similar treatment for the (*S*)-enantiomer yields Eqs. 35-37, where the assumptions described by Eqs. 31 and 32 are also exploited.

$$\widehat{N}_{\mathrm{LC}}^{S} = \frac{\widehat{N}_{\mathrm{T}}^{S} A_{\mathrm{T}}^{S} - \widehat{N}_{\mathrm{LE}}^{S} A_{\mathrm{LE}}^{S}}{A_{\mathrm{LC}}^{S}} \tag{35}$$

$$= \frac{\widehat{N}_{\mathrm{T}}^{R} A_{\mathrm{T}}^{S} - \left(\widehat{N}_{\mathrm{LE}}^{R} + N_{\mathrm{LE}}^{R} - N_{\mathrm{LE}}^{S}\right) A_{\mathrm{LE}}^{S}}{A_{\mathrm{LC}}^{S}} \tag{36}$$

$$= \frac{1}{A_{\mathrm{LC}}^{S}} \left[\frac{N_{\mathrm{LE}}^{R} A_{\mathrm{LE}}^{R} + N_{\mathrm{LC}}^{R} A_{\mathrm{LC}}^{R}}{4 A_{\mathrm{T}}^{R}} A_{\mathrm{T}}^{S} - \left(1 - \frac{N_{\mathrm{LE}}^{S}}{N_{\mathrm{LE}}^{R}}\right) N_{\mathrm{LE}}^{R} A_{\mathrm{LE}}^{S} - \widehat{N}_{\mathrm{LE}}^{R} A_{\mathrm{LE}}^{S} \right] \tag{37}$$

Then, taking the difference and using the known values for the areas and DPPC density ratios leads to Eqs. 38-40.

$$\widehat{N}_{\mathrm{LC}}^{R} - \widehat{N}_{\mathrm{LC}}^{S} = \widehat{N}_{\mathrm{LE}}^{R} \left(\frac{A_{\mathrm{LE}}^{S}}{A_{\mathrm{LC}}^{S}} - \frac{A_{\mathrm{LE}}^{R}}{A_{\mathrm{LC}}^{R}} \right)$$

$$+ N_{\mathrm{LE}}^{R} \left(\frac{A_{\mathrm{LE}}^{R}}{4 A_{\mathrm{LC}}^{R}} - \frac{A_{\mathrm{LE}}^{R}}{4 A_{\mathrm{LC}}^{S}} \frac{A_{\mathrm{T}}^{S}}{A_{\mathrm{T}}^{R}} + \left(1 - \frac{N_{\mathrm{LE}}^{S}}{N_{\mathrm{LE}}^{R}}\right) \frac{A_{\mathrm{LE}}^{S}}{A_{\mathrm{LC}}^{S}} \right) + \frac{N_{\mathrm{LC}}^{R}}{4} \left(1 - \frac{A_{\mathrm{LC}}^{R}}{A_{\mathrm{LC}}^{S}} \frac{A_{\mathrm{T}}^{S}}{A_{\mathrm{T}}^{R}} \right) \tag{38}$$

$$= \widehat{N}_{\mathrm{LE}}^{R} \left(\frac{A_{\mathrm{LE}}^{S}}{A_{\mathrm{LC}}^{S}} - \frac{A_{\mathrm{LE}}^{R}}{A_{\mathrm{LC}}^{R}} \right) + N_{\mathrm{LE}}^{R} \left(\frac{A_{\mathrm{LE}}^{R}}{4 A_{\mathrm{LC}}^{R}} - \frac{A_{\mathrm{LE}}^{R}}{4 A_{\mathrm{LC}}^{S}} \frac{A_{\mathrm{T}}^{S}}{A_{\mathrm{T}}^{R}} + \left(1 - \frac{N_{\mathrm{LE}}^{S}}{N_{\mathrm{LE}}^{R}}\right) \frac{A_{\mathrm{LE}}^{S}}{A_{\mathrm{LC}}^{S}} + \frac{N_{\mathrm{LC}}^{R}}{4 N_{\mathrm{LE}}^{R}} \left(1 - \frac{A_{\mathrm{LC}}^{R}}{A_{\mathrm{LC}}^{S}} \frac{A_{\mathrm{T}}^{S}}{A_{\mathrm{T}}^{R}} \right) \right) \tag{39}$$

$$= 0.60 \widehat{N}_{\mathrm{LE}}^{R} - 0.54 N_{\mathrm{LE}}^{R} \tag{40}$$

This yields a relation for the difference between the POPC densities in the LC domains of the two enantiomeric mixtures which is dependent on the POPC and DPPC density in the (R)-enantiomer LE phase (an analogous expression also exists using the (*S*)-enantiomer LE phase). Given that the LE phase is dominated by POPC, clearly $\widehat{N}_{\mathrm{LE}}^{R} > N_{\mathrm{LE}}^{R}$. This shows that, while the (*S*)-enantiomer domains have greater DPPC density cf. the (R)-enantiomer i.e., $N_{\mathrm{LC}}^{S} > N_{\mathrm{LC}}^{R}$ (from Eq. 29), they have lower POPC density i.e., $\widehat{N}_{\mathrm{LC}}^{R} > \widehat{N}_{\mathrm{LC}}^{S}$. Evidently, therefore, the LC domains in the (*S*)-(R) mixture have a greater excess (higher purity) of DPPC than those in the (R)-(R) mixture.

The other conclusion above where the LC domains in the (*S*)-(R) mixture were shown to possess greater lipid orientational order than those in the (R)-(R) mixture follows well with the observation of greater DPPC excess for the (*S*)-(R) domains as DPPC can pack better. This conclusion can also be furthered by comparing the C1 spectra for both enantiomers, which are shown in Suppl. Fig. 8b. As noted previously, the C1 component largely represents the out-of-plane contribution, thus being dominated by CH_3 resonances. To this end, the spectra from both enantiomers are roughly similar. They do, however, have notable differences. Firstly, the (*S*)-enantiomer clearly has a reduced CH_2 presence, indicated by weaker positive contributions from both the CH_2 symmetric stretch and its Fermi resonance. A reduction in out-of-plane CH_2 could suggest that the tail structure is more 'upright', possessing a lower tilt angle, such that the CH_2 transition dipoles are directed more in-plane. Alternatively, it could also be indicative of a reduction in the density of gauche defects, indicating a more ordered structure.[4] Secondly, whilst only slight, the (*S*)-enantiomer appears to have a lower ratio of the symmetric-to-antisymmetric CH_3 stretches, manifesting mostly as a lower intensity of the latter band. Given that the symmetric CH_3 transition dipole is directed along the angle of the methyl group, and the antisymmetric modes are perpendicular to this, their out-of-plane contributions can be described by cosine and sine functions of the methyl tilt angle. This renders the symmetric-to-antisymmetric ratio in the C1 component (neglecting the sign difference) a cotangent function, indicating that a reduction in this ratio corresponds to an increase in the methyl tilt angle. Whilst these two observations might appear contradictory, it is important to note that the methyl group is generally considered to be directed away from the molecular tilt direction for even chain-length tails.[7] Therefore, for largely upright chains, an increase in methyl tilt angle aligns with a reduced tail tilt angle, indicating that both observations are consistent with each other. Although these observations alone are insufficient for a conclusive determination of the molecular tilt angle, they provide support to the conclusions from the analysis above which suggests an increased order in the (*S*)-enantiomer domains (cf. (R)-enantiomer).

Supplementary References

1. Shen, Y. R. *The principles of nonlinear optics*. (John Wiley & Sons Ltd., New York, 1984).
2. Boyd, R. W. *Nonlinear Optics*. (Elsevier Inc., London, 2020).
3. Morita, A. *Theory of Sum Frequency Generation Spectroscopy*. (Springer, Singapore, 2018).
4. Lambert, A. G., Davies, P. B. & Neivandt, D. J. Implementing the theory of sum frequency generation vibrational spectroscopy: A tutorial review. *Appl. Spectrosc. Rev.* **40**, 103–145 (2005).
5. Wang, H. F., Gan, W., Lu, R., Rao, Y. & Wu, B. H. Quantitative spectral and orientational analysis in surface sum frequency generation vibrational spectroscopy (SFG-VS). *Int. Rev. Phys. Chem.* **24** 191–256 (2005).
6. Qiao, L., Ge, A., Lang, Y. & Ye, S. Oxidative degradation of the monolayer of 1-palmitoyl-2-oleoyl-sn-glycero-3-phosphocholine (POPC) in low-level ozone. *J. Phys. Chem. B* **119**, 14188–14199 (2015).
7. Ma, G. & Allen, H. C. DPPC Langmuir monolayer at the air-water interface: Probing the tail and head groups by vibrational sum frequency generation spectroscopy. *Langmuir* **22**, 5341–5349 (2006).
